# Caloric Restriction Prevents Lead-Induced Oxidative Stress and Inflammation in Rat Liver

**DOI:** 10.1155/2014/821524

**Published:** 2014-02-20

**Authors:** Mustafa Mohammadi, Rana Ghaznavi, Rana Keyhanmanesh, Hamid Reza Sadeghipour, Roya Naderi, Hossein Mohammadi

**Affiliations:** ^1^Drug Applied Research Center, Tabriz University of Medical Sciences, Tabriz 51656-65811, Iran; ^2^Department of Physiology, Faculty of Medicine, Tehran University of Medical Sciences, Tehran 1417613151, Iran; ^3^Dental School, Tabriz University of Medical Sciences, Tabriz 51656-65814, Iran

## Abstract

The aim of the present study is to investigate the effects of caloric restriction on liver of lead-administered rat. Male Sprague-Dawley rats were randomly divided into two groups: Ad libitum fed group (AL, free access to normal rat chow) and caloric restriction group (CR, fed 65% of AL animals' food intake). After 6 weeks, half of the animals of each group were injected lead acetate and the other half were injected saline. Liver tissue samples were collected at the end of the experiments. Glutathione peroxidase (GPx), superoxide dismutase (SOD), malondialdehyde (MDA), and tumor necrosis factor (TNF-**α**) were measured in the tissue extracts. Histological studies were also performed. Our results showed that lead administrations (not saline injections) reduced liver SOD and GPx and increased MDA and TNF-**α** in AL animals, but in the CR animals lead injections did not significantly change the measured parameters. The histological studies supported the biochemical findings. We concluded that 65% CR may prevent lead-induced oxidative stress and inflammation in rat liver.

## 1. Introduction 

Caloric restriction (CR) is referred to reduction of the food intake below ad libitum level without malnutrition. CR is practiced in researches as a method for preventing or delaying the onset of cardiovascular disease, diabetes, and cancer. Experimental work confirming the success of this approach has accumulated over the recent decades [1]. Antioxidative and anti-inflammatory effects of CR have been suggested as one of the mechanisms for health promoting outcomes of CR. CR attenuates age-related vascular oxidative stress and inflammation and improves endothelial function [2]. Clinical significance of CR in preserving *β*-cell function and delaying the onset and treatment of diabetes as a ROS overproduction state is evidenced [3]. Mild fibrotic and inflammatory state of the liver in aged rats can be ameliorated by CR [4].

Lead is a heavy metal with wide toxic effects on liver, brain, heart, and kidney. Although lead toxicity has been relatively controlled in industries, it is still an important health issue in many countries. Local surveillance efforts in Iran prove that lead continues to be found often at toxic levels in the air, soil, and food supply [5]. Lead was reported as the most toxic metal in fresh water of Malaysia [6]. Consumption of vegetables produced on lead-contaminated soils is a health risk in Nigeria [7]. Oxidative stress may play the main role in toxicity of lead due to imbalance in oxidant/antioxidant homeostasis [8].

We aimed the present study to investigate the antioxidative and anti-inflammatory effects of caloric restriction on lead-induced oxidative stress and inflammation in rat liver tissue.

## 2. Materials and Methods

### 2.1. Animals

Twenty-eight male Sprague-Dawley rats weighing 250 ± 10 g were housed individually in regular cages under the controlled environmental conditions (20 ± 2°C and 12 h light–dark cycle) and allowed free access to standard rat chow and tap water. Animal care was in compliance with the guidelines of the Animal and Human Ethical Committee of Tabriz Medical Sciences University.

### 2.2. Experiments Protocol

After an acclimation period, animals were randomly divided into two groups (14 rats in each): Control ad libitum fed group (AL) and caloric restriction group (CR). The animals of AL group were individually housed and had free access to normal rat chow and tap water (ad libitum). The animals of CR group were individually housed and fed with 65% of food intake of AL rats [9]. After six weeks, 7 rats of each group were administered lead acetate (15 mg/Kg body weight solved in 1 mL normal saline, ip, 7 days) [10] and the other 7 rats of each group were injected normal saline (1 mL, ip, 7 days).

During the experiments, mean food consumption of AL rats was measured and 65% of it given to CR rats in daily portions [9]. All rats were weighed weekly. At the end of the experiments, the rats were anaesthetized with ketamine (44 mg/k, ip) and chlorpromazine (30 mg/kg, ip) [11] and the liver tissue samples were collected. The tissue samples were extracted and stored at −70°C for further Glutathione peroxidase (GPx), superoxide dismutase (SOD), malondialdehyde (MDA), and tumor necrosis factor (TNF-*α*) measurements. A part of each tissue sample was fixed in 10% formalin buffer for histological studies.

### 2.3. Measurement of Antioxidant Enzymes Activities

The tissue extracts were used for determination of GPx and SOD. SOD activity was assayed by commercial kit (RANSOD, Randox co., Antrim, UK) according to Delmas-Beauvieux et al. method [12]. This method employs xanthine and xanthine oxidase to generate superoxide radicals which react with 2-(4-iodophenyl)-3-(4-nitrophenol)-5-phenyltetrazolium chloride to form a red formazan dye. The superoxide dismutase activity is then measured by the degree of inhibition of this reaction and was expressed as U/mg Pr.

GPx activity was determined using commercial kit (RANSEL, Randox co., Antrim, UK) according to the method of Paglia and Valentine [13]. Briefly, in the presence of glutathione reductase and NADPH, oxidized glutathione is immediately converted to the reduced form with concomitant oxidation of NADPH to NAD+. The decrease in absorbance at 340 nm (37°C) was measured. GPx concentration was calculated by the related formula and expressed as U/mg Pr.

### 2.4. Lipid Peroxidation Study

MDA as the end-product of lipid peroxidation was measured in the tissue extracts according to the Esterbauer and Cheeseman method [14]. MDA reacts with thiobarbituric acid and produces a pink pigment that has maximum absorption at 532 nm.

### 2.5. Assay of Inflammatory Cytokine

The concentration of TNF-*α* as inflammation marker was determined by an enzyme-linked immunosorbent assay (ELISA) in 450 nm wave length using commercial rat TNF-*α* assay kit (eBioscience, San Diego, CA, USA). The assays were carried out according to the manufacturers' instructions.

### 2.6. Histological Studies

The liver tissue samples were fixed in 10% formalin buffer and embedded in paraffin. The sections of the tissues (4 *μ*m) were stained with hematoxylin and eosin. The stained sections were evaluated in ×400 magnifications for the presence of congestion, necrotic changes, and leukocyte infiltration as oxidative stress and inflammation histological signs.

### 2.7. Statistics

All numerical data are expressed as mean ± SEM. The data was subjected to ANOVA and Tukey's test. A *P* value less than 0.05 was considered statistically significant.

## 3. Results

### 3.1. Body Weight

**Figure 1:**
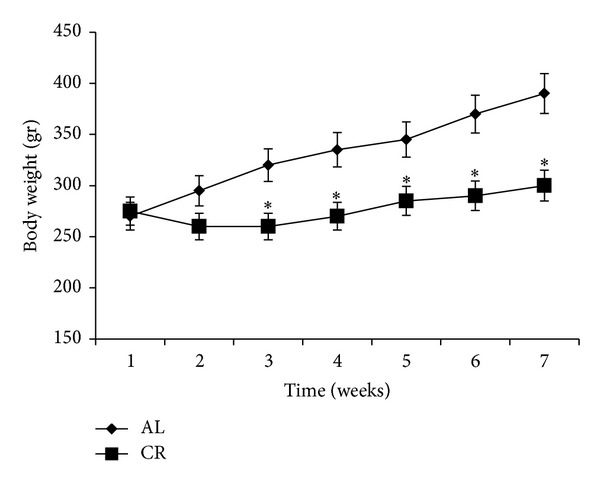
Body weight in the ad libitum (AL) and caloric restriction (CR) groups during the experiments. CR rats were given 65% of AL animals food intake. After 6 weeks, lead (15 mg/Kg body weight, ip, 7 days) or saline was injected (AL + saline and CR + saline not shown). CR rats had lower weight from the 2nd week to the end of the experiments compared to the AL rats (*P < 0.05 compared to the AL rats). The data are presented as means ± SEM.

AL rats gained weight continuously during the experiments (total mean weight gain of 120 gr in 7 weeks). CR rats lost weight in the first week, but started slight weight gain from the third week (total mean weight gain of 25 gr in 7 weeks). CR rats had lower weight from the 2nd week to the end of the experiments compared to the AL rats (*P* < 0.05, Figure 1). Saline or lead injections in the last week had no significant effect on body weight.

### 3.2. Antioxidant Enzymes

**Figure 2:**
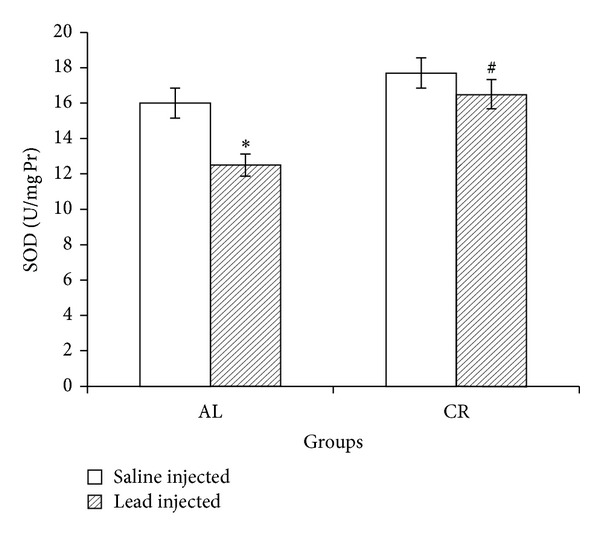
Liver superoxide dismutase (SOD) in ad libitum (AL) and caloric restriction (CR) groups after lead or saline administrations. Seven days of lead injections (15 mg/Kg body weight, ip) to AL animals caused significant reduction of liver SOD (*P < 0.05 compared to the saline-injected AL); in CR animals SOD changes after the same dose lead administration were not significant. Liver SOD content was significantly higher in lead-administered CR compared to lead-administered AL (^#^ *P* < 0.05 compared to the lead-injected AL). The data are presented as means ± SEM.

**Figure 3:**
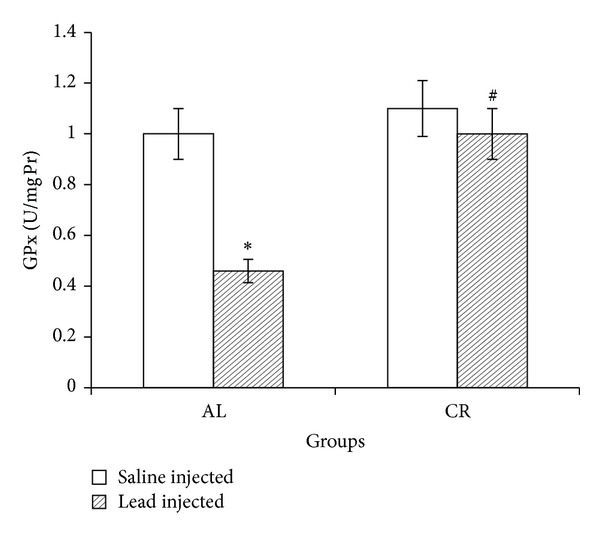
Liver glutathione peroxidase (GPx) in ad libitum (AL) andcaloric restriction (CR) groups after lead or saline administrations. Seven days of lead injections (15 mg/Kg body weight, ip) to AL animals caused significant reduction of liver GPx (*P < 0.05 compared to the saline-injected AL); in CR animals GPx changes after the same dose lead administration were not significant. Liver GPx content was significantly higher in lead-administered CR compared to lead-administered AL (^#^ *P* < 0.05 compared to the lead-injected AL). The data are presented as means ± SEM.

Lead administration in AL group caused significant reduction in liver SOD and GPx activities compared to saline-administered animals while, in CR group, lead-induced SOD and GPx changes were not significant compared to the saline-injected CRs (Figures 2 and 3).

### 3.3. Lipid Peroxidation Study

**Figure 4:**
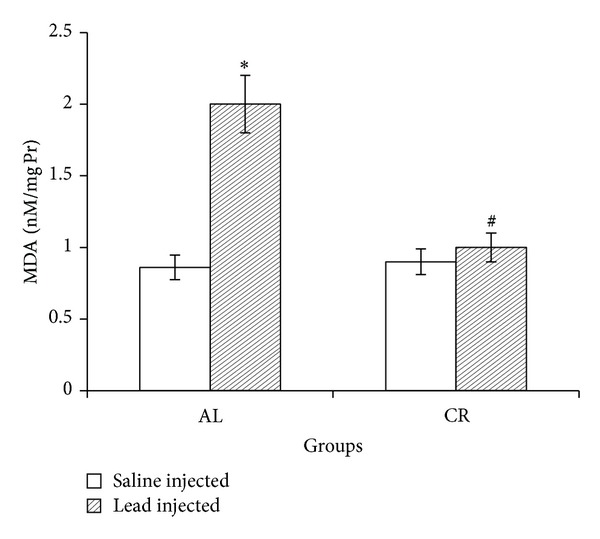
Liver malondialdehyde (MDA) in ad libitum (AL) and caloric restriction (CR) groups after lead or saline administrations. Seven days of lead injections (15 mg/Kg body weight, ip) to AL animals caused significant raise of liver MDA (*P < 0.05 compared to the saline-injected AL); in CR animals MDA changes after the same dose lead administration were not significant. Liver MDA content was significantly lower in lead-administered CR compared to lead-administered AL (^#^ *P* < 0.05 compared to the lead-injected AL). The data are presented as means ± SEM.

Lead administration in AL group increased liver MDA content when compared to saline-administered rat. In CR group, lead-induced MDA changes were not significant (Figure 4).

### 3.4. Inflammatory Cytokine

**Figure 5:**
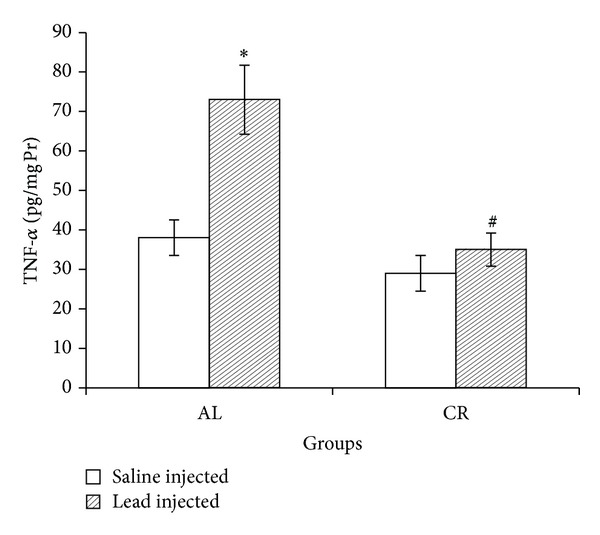
Liver tumor necrosis factor-*α* (TNF-*α*) in ad libitum (AL) and caloric restriction (CR) groups after lead or saline administrations. Seven days of lead injections (15 mg/Kg body weight, ip) to AL animals caused significant raise of liver TNF-*α* (*P < 0.05 compared to the saline injected AL); in CR animals TNF-*α* changes after the same dose lead administration were not significant. Liver TNF-*α* was significantly lower in lead-administered CR compared to lead-administered AL (^#^ *P* < 0.05 compared to the lead-injected AL). The data are presented as means ± SEM.

Mean liver TNF-*α* level was significantly increased after 7 days of lead injections in AL group. Lead administration in CR rats did not significantly change liver TNF-*α* (Figure 5).

### 3.5. Histological Studies

**Figure 6:**
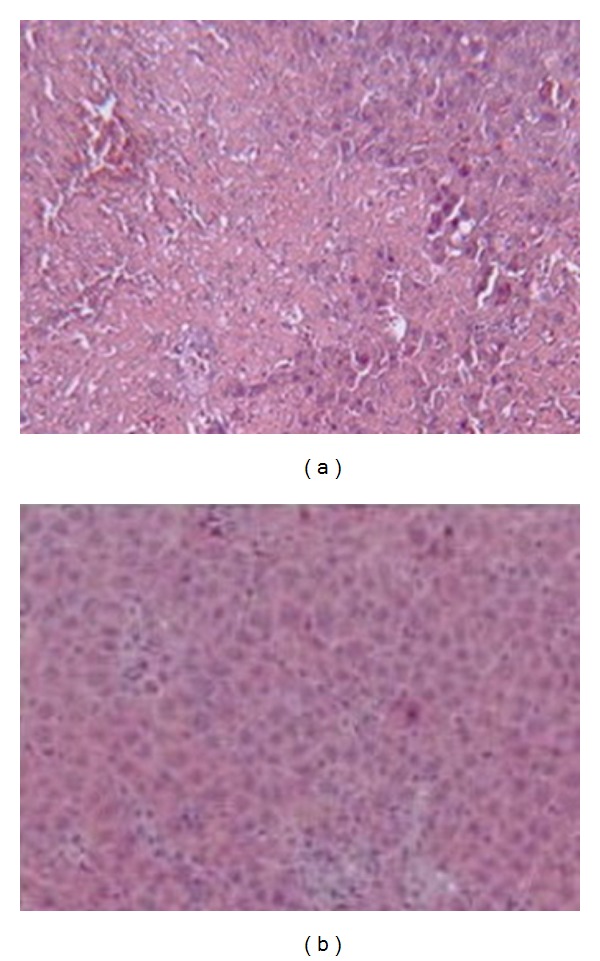
Histological evaluations of the liver sections. The liver sections of the lead-treated ad libitum fed animals (a) showed clear signs of necrosis and inflammation, while there was trend for improvement in the livers of the lead-treated animals in caloric restriction group (b) showing normal histology (×400).

Lead administration in AL group caused clear necrotic changes, congestion, and leukocyte infiltration in the liver tissue. The sections from the lead administered CR rats displayed minimal/no changes (Figure 6). Liver sections in saline injected animals in both groups showed normal histology (not shown).

## 4. Discussion

According to the results of the present study, lead administration induced oxidative stress (decreased SOD and GPx and increased MDA) and inflammation (increased TNF-*α*) in liver. A period of 6 weeks of CR prevented lead-induced liver oxidative stress and inflammation. The histological studies supported our findings.

Some previous studies have suggested the same protective role for CR against oxidative and inflammatory stresses. Singh et al. have shown that CR had the potential to retard age-associated oxidative molecular damage to proteins of brain tissue and preserved cognitive and motor performance [15]. Mattson and Wan indicated increased resistance of heart cells to ischemic injury in experimental models of myocardial infarction. The beneficial effects of CR result from reduced oxidative damage and increased cellular stress resistance. Interestingly, cellular and molecular effects of CR on the cardiovascular system are similar to those of regular physical exercise [16].

Liver tissue is one target of CR effects. It is demonstrated that progression of liver injury and death in toxin-injected CR rats was at a lower rate compared to AL animals. CR rats showed more efficient tissue and DNA repair [1]. CR also delays hepatic dysfunction by profound positive effects on the hepatic microsomal levels and activities of cytochrome P-450s [17]. Horrillo et al. reported that CR ameliorated fibrotic and inflammatory changes in the liver of aged rats [4]. Our findings supported protective effects of CR against oxidative and inflammatory stresses in the liver.

Lead is a heavy metal which is used in more than 900 industries [5]. The phasing out of leaded gasoline for transportation and the removal of lead from paint has resulted in substantial lowering of mean blood lead levels. However, because lead is a persistent metal, it is still present everywhere in the environment—in water, soil, and imported products manufactured with lead [18]. Exposure to lead produces various deleterious effects on the liver, kidney, and central nervous system, mainly through increased oxidative stress. Different antioxidants have been successfully used in previous studies to prevent or treat systemic lead toxicity, including vitamins, flavonoids, alpha lipoic acid, and herbal antioxidants like garlic [19]. The results of the present study indicated that CR with known antioxidant effect prevents lead-induced toxicity in liver.

## 5. Conclusion

The results of the present study demonstrated CR preventive role in lead-induced oxidative stress and inflammation in rat liver. We suggest that CR, beside its other beneficial effects, may be considered as a protective protocol against lead-induced oxidative stress and inflammation in liver tissue.
